# Hall measurements reveal band-like transport in high-mobility solution-processed organic semiconductor films

**DOI:** 10.1093/nsr/nwae266

**Published:** 2024-08-08

**Authors:** Reverant Crispin, Igor Zozoulenko

**Affiliations:** Laboratory of Organic Electronics, Department of Science and Technology, Linköping University, Sweden; Laboratory of Organic Electronics, Department of Science and Technology, Linköping University, Sweden

**Figure 1.**
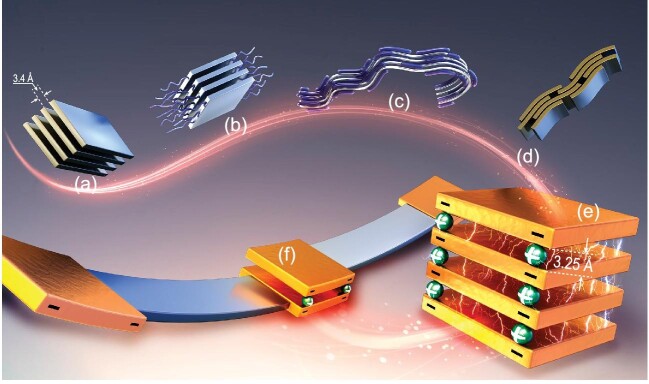
(a) Molecular single crystal, (b) conjugated molecule carrying side chains, (c) conjugated polymer carrying side chains, (d) local aggregation of chains forming a percolation path, (e) perylene diimide (PDI) dianion crystal and (f) imaginary PDI dimerization to attach polymer chains.

Organic semiconductors, composed of conjugated molecules or polymers, can be designed with specific chemical compositions, crystalline structures, nano-morphologies and electronic properties using extensive chemical synthesis tools. High charge-carrier mobility, which is crucial for device performance in transistors, organic light emitting diodes (OLEDs) and solar cells, is influenced by the overlap between the π-orbitals of adjacent molecules. While single crystals (Fig. 1a) provide valuable insights into anisotropy and transport mechanisms, they are impractical for complex circuits. The reduction of impurities and traps has led to encouraging results, with mobilities surpassing that of amorphous silicon. For example, rubrene shows reproducible mobility of >15 cm² V^−1^ s^−1^ due to its specific slipped π-stack packing [1]. Yet, the advantage of organic semiconductors lies in their ability to form thin layers via low-temperature solution processing, enabling cost-effective printing techniques such as inkjet and roll-to-roll processing.

Several strategies have been explored to enhance charge-carrier mobility in soluble organic semiconductors. Conjugated molecules have been modified with side chains to promote solubility and self-organization (Fig. 1b), resulting in improved π–π stacking and mobilities. Examples include herringbone-stacked alkylated thienoacene-based materials [2] and their liquid crystalline derivatives [3], which achieve charge-carrier mobilities of >10 cm² V^–1^ s^−1^ and exhibit transport characteristics similar to those of rubrene, including an ideal Hall effect. A challenge with solution-processed small molecules is their polycrystalline nature, requiring extensive morphology optimization to approach the performance of single crystals [4]. Polymers facilitate pinhole-free thin films and the introduction of side chains along the polymer backbone aids in controlling processability and nanomorphology during device manufacturing (Fig. 1c) [5]. Another strategy involves allowing macroscopic disorder in the polymer layer while promoting local chain aggregation (Fig. 1d) through the formation of flat polymer structures [6], as seen with donor–acceptor (D–A) copolymers that often exhibit mobilities of >1 cm² V^−1^ s^−1^ [7].

High mobility in semiconductor materials is usually associated with band-like (metallic) transport, in which electrons are delocalized over the crystal and move freely in conduction (or valence) bands. On the contrary, at elevated temperatures or in less ordered organic semiconductors, electron transport is dominated by hopping between localized states. One of the most effective tools for elucidating the characteristics of electron transport in semiconductors is the Hall effect, which represents evidence of electron delocalization and band-like transport. This is because the Lorentz force responsible for the Hall effects acts on carriers with a well-defined drift velocity (in band-like states) and not on localized carriers supporting the hopping transport.

In a recent study led by Prof. Yuguang Ma, solution-processed perylene diimide (PDI) dianion films displayed the Hall effect, which represents evidence of band-like transport with high Hall mobility of 0.5 cm² V^–1^ s^−1^ at room temperature and an electrical conductivity of 17 S cm^–1^ [8]. The reason for such high mobility was attributed to the enhanced interaction between π-stacked aggregates leading to surprisingly short intermolecular distances of 3.25 Å when the molecules were doped with two electrons per molecule and balanced with ammonium cations (Fig. 1e). In a film with neutral molecules, the intermolecular distance is larger and similar to what is typically found in other crystals and aggregates made of conjugated molecules (∼3.4 Å). Density functional theory calculations showed enhanced localization on the flat PDI dianions and increased overlap of the π-orbitals. This leads to the formation of so-called ‘pancake bonds’, which have stronger interactions than typical π–π interactions and whose strength is comparable to that of covalent bonding.

This finding opens up new routes for the design of high-mobility semiconductors by creating other flat organic dyes forming strong π–π interactions upon n-doping. Moreover, we could speculate on and wonder whether a conjugated polymer chain could potentially carry two end groups composed of PDI molecules, which, once reduced, could form covalent anchor points between the chains (Fig. 1f). If this is possible, then the slow interchain charge-carrier hopping that currently limits transport in conjugated polymer films could be replaced by fast transport through interchain delocalized states. Ultimately, this represents an intermediate step towards a 3D conjugated polymer structure that would resemble inorganic semiconductors, as weak intermolecular interactions are absent.
